# Differential Expression of Tie2 Receptor and VEGFR2 by Endothelial Clones Derived from Isolated Bovine Mononuclear Cells

**DOI:** 10.1371/journal.pone.0053385

**Published:** 2012-12-31

**Authors:** Una Adamcic, Alexander Yurkiewich, Brenda L. Coomber

**Affiliations:** Department of Biomedical Sciences, Ontario Veterinary College, University of Guelph, Guelph, Ontario, Canada; University of Kansas Medical Center, United States of America

## Abstract

The purpose of these experiments was to evaluate the expression of endothelial markers, such as Tie2 and VEGFR2 in endothelial cells derived from blood mononuclear endothelial progenitor cells. Bovine mononuclear cells were isolated using separation by centrifugation and were grown in endothelial specific media supplemented with growth factors. Isolation of the whole cell population of mononuclear cells (MNC) from bovine peripheral blood gave rise to progenitor-like cells (CD45^−^) that, although morphologically similar, have different phenotypes revealed by expression of endothelial specific markers Tie2 and VEGFR2. Plating of MNCs on collagen and fibronectin gave rise to more colonies than non-coated dishes. Occasional colonies from MNC isolations had a mural cell phenotype, negative for Tie2 and VEGFR2 but positive for smooth muscle actin and PDGFRβ. Although cells expressing high levels of VEGFR2 and low levels of Tie2, and vice versa were both able to form cords on Matrigel, cells with higher expression of Tie2 migrate faster in a scratch assay than ones with lower expression of Tie2. When these different clones of cells were introduced in mice through tail vein injections, they retained an ability to home to angiogenesis occurring in a subcutaneous Matrigel plug, regardless of their Tie2/VEGFR2 receptor expression patterns, but cells with high VEGFR2/low Tie2 were more likely to be CD31 positive. Therefore, we suggest that active sites of angiogenesis (such as wounds, tumors, *etc.*) can attract a variety of endothelial cell precursors that may differentially express Tie2 and VEGFR2 receptors, and thus affect our interpretation of EPCs as biomarkers or therapies for vascular disease.

## Introduction

Angiogenesis was long thought to represent the principle mechanism used for neovascularization [1], until the last decade or so, when focus shifted towards studying the role of bone marrow derived cells. In particular, a subset of these cells called endothelial progenitor cells (EPCs), are involved in adult neovascularization [2]. To date, these progenitors have been shown to have an ability to mobilize from the bone-marrow into peripheral circulation, home to sites of angiogenesis, differentiate into mature endothelial cells and incorporate into the vasculature at the sites of ischemia, tumor formation and myocardial infarction [3]–[7].

Defining the precise role and contribution to vasculature of these EPCs has been an intense area of research due to important implications for novel anticancer therapy, ischemia, wound healing, and tissue engineering [8]–[10]. In fact, transplantation of isolated and *ex vivo* expanded human EPCs improved blood flow recovery and capillary density in several animal hind-limb ischemia models [9] and improved ischemic heart conditions [10]–[11]. Clinical trials using autologous progenitor cell transplantation are underway, showing great promise for patients with ischemic limbs as a consequence of peripheral arterial disease [12]–[15].

However, the exact identity of EPCs and the nature of the cells they give rise to is conflicting, in part due to the highly variable approaches, different cell sources, methods of cell purification, and animal models employed [16]. Lack of a distinct EPC marker makes routine identification of these cells a challenge as there are important phenotypical and functional overlap between EPCs, haematopoietic cells and mature endothelial cells [17]–[22]. For instance, haematopoietic-derived cells such as monocytes, granulocytes and even haematopoetic stem/progenitor cells have been shown to co-express a host of similar surface markers as endothelial cells [20]–[22]. Like EPCs, these bone marrow derived cells are also involved in vascular repair, making them difficult to discriminate from each other [19].

Variability in identification and characterization of EPCs by different research groups also arises due to differences in methodology used to isolate these cells. Plating of peripheral blood mononuclear cells onto fibronectin in culture medium enriched with endothelial specific growth factors gives rise to EPCs, characterized by expression of mature endothelial markers [23]. However, others have shown that similarly isolated and cultured adherent cells expressing the above antigens may also co-express macrophage markers [22]. In contrast, plating of peripheral blood mononuclear cells on type I rat tail collagen yields early arising colonies that display limited proliferative potential, as well as late arising colonies with high proliferative potential [17]–[18]. Researchers have also collected non-adherent cells and replated them on fibronectin, producing two types of colonies- endothelial cell colony forming units (EC-CFUs) and blood outgrowth endothelial cells (BOECs) [18], [20]. Use of monoclonal antibodies and fluorescence activated cell sorting to isolate and enumerate cells that express particular markers of interest is also employed [21] but the vascular phenotype of such sorted cells is not always confirmed.

Here, we investigated the molecular and functional features of bovine endothelial cells derived from circulating peripheral blood mononuclear cells, to determine if they can give rise to endothelial linage, and if their differentiated phenotype can be influenced by maturation on different extracellular matrices. In addition, we investigated the expression profiles of their endothelial markers, and used *in vitro* and *in vivo* assays to determine if differential expression of endothelial receptor tyrosine kinases influenced angiogenic capacity. These studies have important implications in our understanding of EPCs, and how they may impact therapeutic applications for vascular disease, or cancer therapeutics designed to target specific endothelial markers.

## Materials and Methods

### Ethics Statement

All procedures involving animals described below were done according to the guidelines and recommendations of the Canadian Council on Animal Care and approved by the University of Guelph Local Animal Care Committee. Cows were housed in tie-stalls in the OVC large animal wards; this facility is inspected and approved by the College of Veterinarians of Ontario and the CCAC, and accredited by the American Veterinary Medical Association. Cows were returned to the teaching herd after blood sampling. Mice were group housed (5 per cage) in the barrier facility of the University of Guelph Central Animal Facility. Mice had access to food and water ad libitum, and cages were provided with nesting cubes and mouse huts. Mice were identified via ear punch marks, and were given analgesia for this procedure. Mice were anesthetized with Avertin prior to subcutaneous injection. According to CCAC guidelines, local ACC approval was not required for collection of slaughterhouse material.

### Buffy-Coat Preparation

Peripheral blood samples (approximately 100 mL per isolation) were collected from tail veins of healthy adult female Holstein cows. Blood was collected into vacutainers (BD Biosciences, Mississauga, ON, Canada) containing acid citrate dextrose (ACD) solution. Samples were centrifuged at 1200×g for 20 minutes to separate blood. Buffy-coat mononuclear cells (MNCs) were collected and any red blood cells were lysed with 4 M Ammonium Chloride Solution for 4 minutes. Cells were spun at 350×g for 4 minutes and the pellet washed twice with HBSS (Invitrogen, Burlington, ON, Canada). Finally, the pellet was resuspended in complete EGM-2MV (supplemented with 2% fetal bovine serum, VEGF, IGF, FGF, EGF, hydrochortisone, gentamicin and ascorbic acid) (Lonza, Basel, Switzerland). Mononuclear cells were counted and 4% Trypan Blue (Sigma-Aldrich, Oakville, ON, Canada) was also used to determine cell viability, and cells were plated and cultured in supplemented EGM-2MV medium.

### Culture of Mononuclear Cells to Obtain EPC Derived Colonies

To investigate whether plating of isolated blood mononuclear cells onto different extracellular matrices had an effect on the number of EPC derived colony “islands” that formed, freshly isolated cells were seeded at a density of 5000 viable cells/cm^2^ into separate plates pre-coated using fibronectin (Sigma-Aldrich) or type IV collagen (BD Biosciences, Mississauga, ON, Canada), or left uncoated to serve as a control. Collagen was prepared by adding 4.2 mL of type IV rat tail collagen to 1.2 mL of 7.5% Sodium Bicarbonate Solution (Invitrogen, Burlington, ON, Canada) and 0.6 mL of 10X Minimal Essential Medium (Invitrogen). One mL of the solution was added to each well of a 6-well plate. The plate was incubated for 20 minutes at room temperature to allow the collagen to solidify into a gelatinous state. Similarly, plates were pre-coated with fibronectin (Sigma-Aldrich) that was diluted using sterile PBS solution (Sigma-Aldrich) to 10 µg/mL. Thirty minutes after the solution was added, PBS residue was removed, and plates were washed twice with fresh sterile PBS. All plates were maintained at 37°C, 5% CO_2_, in a humidified atmosphere. Media was changed every 3–4 days and non-adherent cells were discarded. Mononuclear colony “islands” were enumerated under 10X magnification with inverted phase contrast microscopy. In a similar experiment, we investigated whether the removal of non-adherent cells and the media 3, 5 or 7 days after incubation of mononuclear cells in normoxia, or 7 days after incubation in hypoxia (<0.01% O_2_), affected the final number of EPC colony “islands” obtained.

Seeding of 40×10^6^ viable mononuclear cells/100 mm culture dish was more efficient and therefore this protocol was used in further isolations. Mononuclear cells were seeded onto collagen, fibronectin or non-coated plates. Once media was replaced 3 days after the initial plating, colonies appeared anywhere from 4–21 days later. While the colonies were still small enough to remain physically distinct, they were collected from the original plate using cloning disks (Sigma-Aldrich) dipped in trypsin, and seeded into separate wells of non-coated 48-well tissue culture plates and grown in complete EGM-2 MV until confluence was reached. At this point, clones were expanded for further characterization. Primary bovine aortic endothelial cells (BAEC) were isolated from aorta collected at slaughter from adult cattle from a local abattoir (Better Beef Inc., which granted permission for collection of tissue). Cells were characterized as previously reported [24] and used in experiments as control endothelial cells.

### Western Blot Analysis

In order to characterize the expression patterns of the endothelial clones obtained, cells were grown to confluence and lysed using cell lysis buffer, containing 20 mM Tris-HCl (pH 7.5), 150 mM NaCl, 1 mM Na_2_EDTA, 1 mM EGTA, 1% Triton-X, 2.5 mM sodium pyrophosphate, 1 mM beta-glycerophosphate, 1 mM Na_3_VO_4_, 1 µg/mL leupeptin, 1 mM PMF and 2 µg/mL aprotinine (Cell Signaling Technology, Danvers, MA, USA). Proteins were separated using SDS-PAGE on 7.5% polyacrylamide gel, transferred to a PVDF membrane (Roche, Laval, Quebec, Canada) using wet transfer, blocked using 5% milk for 1 hour and probed overnight using either goat anti-CD31 (1∶1000, Santa Cruz, Santa Cruz, CA, USA), rabbit anti-VEGFR2 (1∶1000, Cell Signaling Technology), mouse anti-N-cadherin (1∶2500, BD Biosciences), mouse anti-α-smooth muscle actin (1∶1000, Sigma-Aldrich), rabbit anti-PDGFRβ (1∶1000, Cell Signaling Techlonogy), mouse anti-Tie2 (1∶1000, Cell Signaling Technology) or mouse anti-VEGFR1 (1∶1000, AbCam, Cambridge, MA, USA). Mouse anti-α-tubulin (1∶400,000, Sigma-Aldrich) was used for normalization purposes. Appropriate anti-mouse (1∶40,000, Sigma-Aldrich), anti-rabbit (1∶10,000, Sigma-Aldrich) or anti-goat (1∶40,000, Sigma-Aldrich) secondary POD antibodies were used. Bands were visualized using Chemiluminescence detection kit (Roche), exposed to X-ray film and quantified by densitometry. Tubulin signal was used for normalization purposes.

### Characterization of Angiogenic Capacity *in vitro*

For *in vitro* cord formation experiments 100 µL of Matrigel (B&D Biosciences) was plated into 24-well tissue culture plate and solidified at 37°C for 30 minutes. Representative clones (chosen based on variable VEGFR2 and Tie2 expression) were trypsinized, and plated at 15,000 cells per well in endothelial specific media EGM-2MV (Lonza). Cords were observed and images taken 6 h post plating. For scratch assays, representative clones (chosen based on variable VEGFR2 and Tie2 expression) were plated into 6 well culture dishes and cultured until confluent. A lateral wound was created in the endothelial cell monolayer using a sterile pipette tip, wells were washed twice with sterile PBS (Sigma), and fresh EGM-2MV media was added. Closing of the wound was observed by phase contrast microscopy and images were captured 24 h later.

### Fluorescent Labeling Using Cell Tracker Probes

To be able to visualize injected cells, clones of EPC derived endothelial cells (chosen based on variable VEGFR2 and Tie2 expression) were labeled in vitro using the CellTracker Red CMPTX (577/602 nm) Kit (Invitrogen) to produce red fluorescent cells. Immediately prior to labeling, CellTracker Red CMPTX fluorescent probe was diluted in PBS to a previously optimized working concentration of 5 µM. Confluent cells were washed in PBS and exposed for 15 minutes to pre-warmed diluted fluorescent probe at 37°C. Solution was then removed and replaced with DMEM (Sigma-Aldrich) supplemented with 10% fetal bovine serum, 50 µg/mL gentamicin and 1 mmol/L sodium pyruvate for at least 30 minutes. Cells were trypsinized, counted and resuspended to a concentration of 2×10^6^ cells/50 µL of sterile 0.1% BSA/PBS solution.

### *In vivo* Angiogenesis Assay

Twenty-five female athymic nude mice (8 weeks of age; Charles River Canada) were injected subcutaneously with 500 µL of ice-cold Matrigel (BD Biosciences) premixed with 5 µg/mL of recombinant murine VEGF (Roche) to initiate a strong angiogenic response. Mice were anesthetized with tribromoethanol (Avertin; 0.5 mL i.p.) prior to subcutaneous injection. After 21 days, each mouse was injected into the tail veil with 50 µL of cell suspension containing 2×10^6^ CellTracker Red CMPTX labeled cells. Each fluorescently labeled endothelial clone was injected into 3 mice; 5 different clones with variable VEGFR2 and Tie2 expression patterns were injected. After 3 days, mice were euthanized by CO_2_ asphyxia and cervical dislocation. Matrigel plugs were removed as well as pieces of lung, liver and kidney, placed in OCT cryomatrix and snap frozen in liquid nitrogen for further analysis.

### Visualization of Injected Cells

Ten micron thick cryosections were obtained from Matrigel plugs, lung, liver and kidney. Sections were briefly air dried and fixed in 4% paraformaldehyde for 15 minutes at room temperature, followed by washing in PBS. To stain blood vessels, fixed cryosections were blocked for 30 minutes using Dako protein block (Dako, Carpinteria, CA, USA) and incubated for 1 h with rat CD31 antibody (dilution 1∶100) (Hycult Biotechnology, Uden, NL). After washing in PBS sections were incubated in goat anti-rat FITC secondary antibody (dilution 1∶200) (Jackson ImmunoResearch Laboratories, West Grove, PA, USA) for 30 minutes. To visualize the cellular nuclei, sections were stained with DAPI for 2 minutes and mounted using Aquapolymount (Polyscience, Warrington, PA, USA). Slides were visualized using an epifluorescent microscope (Olympus) and several fields/section were randomly chosen for vessel evaluation. Images were quantified to determine the proportion of CMPTX cells positive for CD31 endothelial marker.

### Statistical Analysis

Calculation of preliminary summary statistics such as mean, standard deviation and standard error as well as graphing all the data was completed using Microsoft Excel (Microsoft). ANOVA was performed to determine the significance within and between groups (p<0.05).

## Results

### Phenotypic Differences in Endothelial Cells Arising from Bovine Peripheral Blood Mononuclear Cells

**Figure 1:**
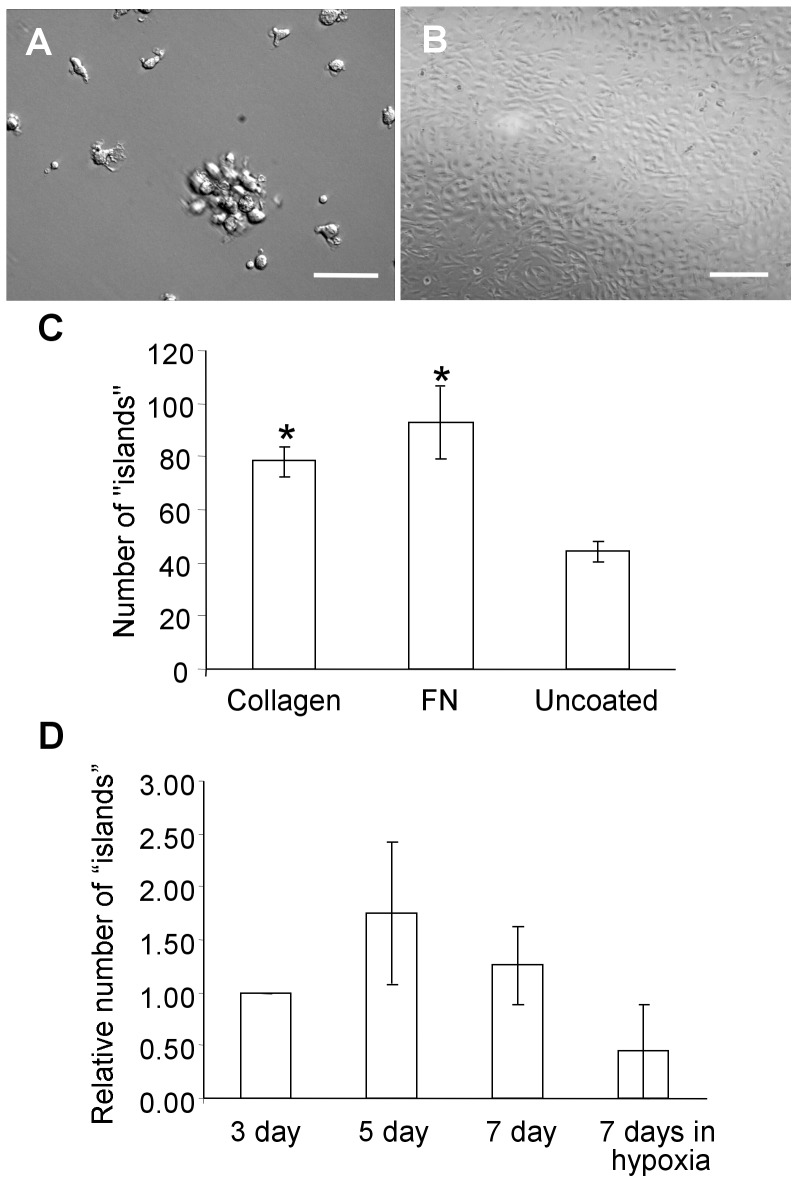
Phenotype of cells arising from peripheral blood mononuclear cells. Phase contrast images of cell “islands” (**A**) and an endothelial clone (**B**). *Scale bar  = 100 µm* (**C**) Effect of different plating conditions on colony “island” formation of bovine mononuclear cells – the number of colony “islands” counted on collagen and fibronectin (FN) coated plates was statistically greater (*p<0.05) than from non-coated (control) plates. (**D**) The number of colony “islands” observed in non-coated plates where non-adherent cells were removed 3, 5 or 7 days after incubation in normoxic conditions and 7 days after incubation in hypoxic conditions. No statistically significant difference (p>0.05) was observed.

Adherent cells yielded colonies of two distinct phenotypes that appeared between days 4–21: mononuclear cell “islands” resembling CFU-EC colonies, and characteristic cobblestone endothelial colonies (Figure 1A&B). The phenotype of these colonies did not appear to depend on either the time to outgrowth (4 vs. 21 days) or the matrices (collagen, fibronectin or none) on which they were grown. Although colonies readily grew on all plates – with no coating, with fibronectin or collagen – the number of mononuclear cell “islands” obtained from fibronectin and collagen coated plates was significantly higher than from plates that were not coated with any extracellular matrix (Figure 1C). CFU-ECs-like cell islands failed to form secondary colonies when transferred to a 48 well plate using cloning disks and were therefore unable to be studied further. Colonies with endothelial phenotype readily survived subcloning and, could be expanded and further passaged.

We found that removal of non-adherent cells 3, 5 and 7 days after initial seeding of mononuclear cells did not significantly increase the number of mononuclear cell “islands” and endothelial colonies observed (Figure 1D). Some studies suggested that increased number of EPC colonies arise in cultures that are hypoxic pre-conditioned [25]. However, maintenance of plates for 7 days in hypoxic conditions after plating did not produce any significant difference in the number of attached cellular “islands” (Figure 1D).

### Characterization of Endothelial Colonies Reveals Differential Expression of Receptor Tyrosine Kinases

**Figure 2:**
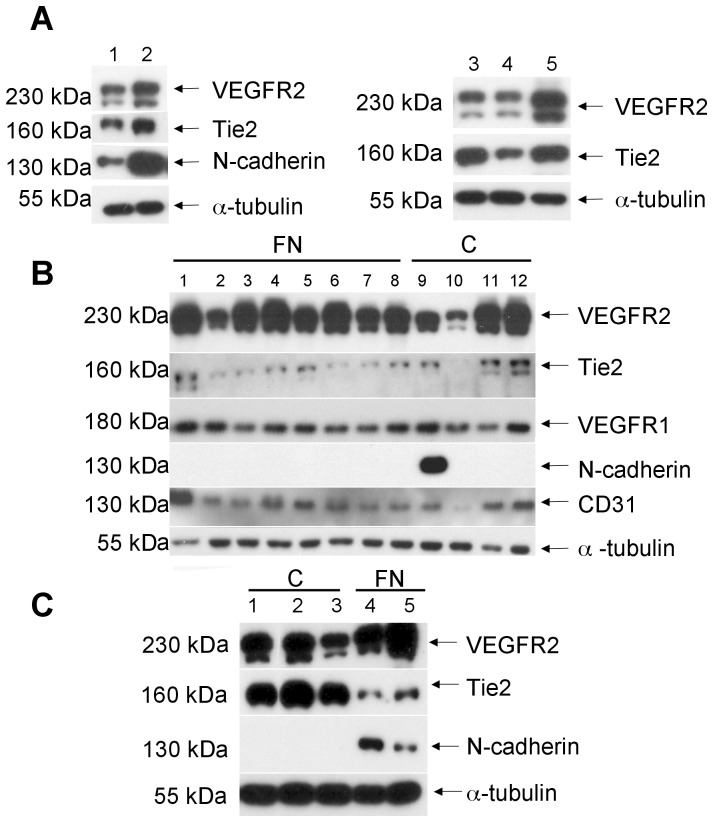
Western blot analysis of isolated bovine clones grown on different extracellular matrices. (**A**) Composite blots of 5 different clones grown on non-coated plates (control conditions) shows clear expression of endothelial markers. Blots also demonstrate that some of the clones express N-cadherin. (**B**) A composite blot of 12 different clones from the same bovine mononuclear cell isolation as in (**A**) but grown on fibronectin (FN) or collagen (C) coated plates. (**C**) Composite blot of a different bovine mononuclear cell isolation showing phenotype of clones obtained from collagen (C) and fibronectin (FN) coated plates.

Western blotting was chosen for characterization of resulting EPC derived clones as the antibodies available had not been validated for flow cytometery, and to allow for evaluation of intracellular proteins such as smooth muscle actin and tubulin. Blots revealed that EPC derived endothelial clones were expressing endothelial markers in a heterogeneous fashion (Figure 2A–C). Only some endothelial clones expressed N-cadherin but the significance of this has yet to be determined. Plating of mononuclear cells on fibronectin, collagen or uncoated dishes did not consistently impact receptor tyrosine kinase expression patterns of the resultant endothelial clones (Figure 2B&C).

**Figure 3:**
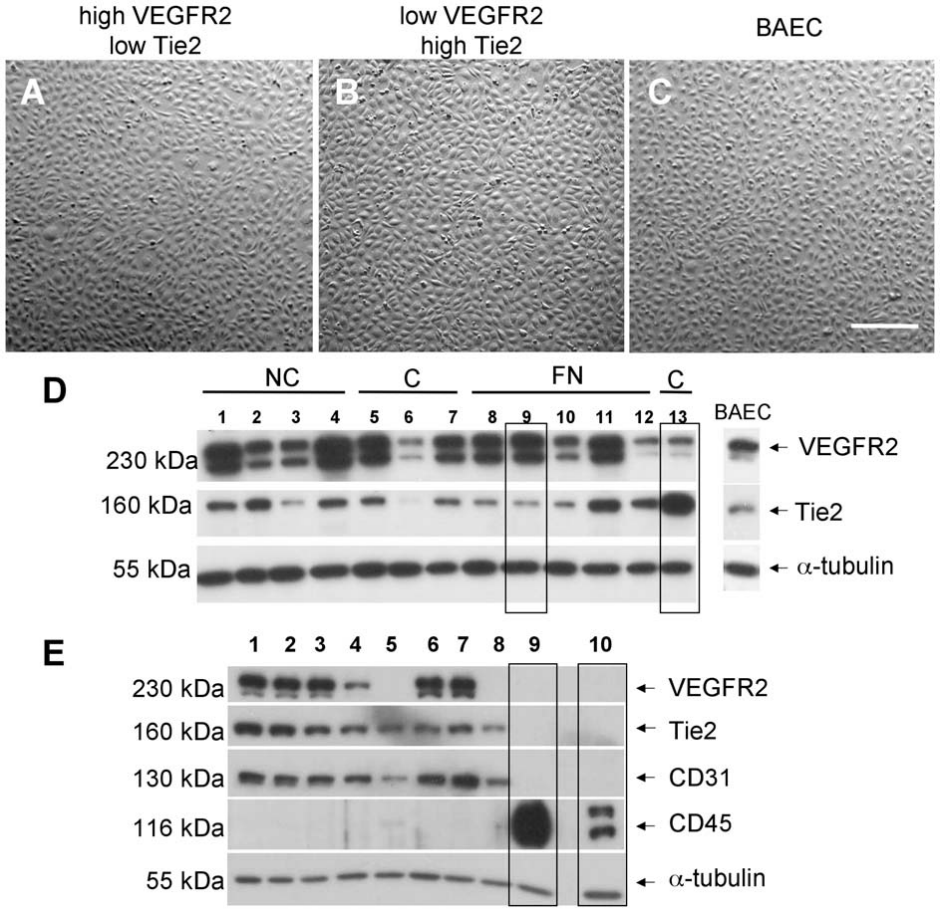
Phenotype of clones with a differential expression of Tie2/VEGFR2 receptors. (**A–C**) Clones with high or low Tie2 and/or high and low VEGFR2 do not reveal any obvious differences in monolayer morphology between one another and compared to BAECs, by phase contrast microscopy. *Scale bar  = 100 µm* (**D**) Western blot analysis of representative clones isolated from MNCs grown on either collagen (C) or fibronectin (FN) matrices or non-coated plates (NC) revealed highly differential expression of VEGFR2 and Tie2; proteins were normalized to α-tubulin. Representative clones are marked with boxes – clone 9 has high VEGFR2 and low Tie2 expression and clone 13 has high Tie2 and VEGFR2 expression, relative to one another and to BAECs. (**E**) Random clones from several isolations were stained for the expression of monocyte surface antigen CD45, revealing that all the clones are negative for this marker. Lanes 9 and 10 depict positive controls for CD45: BJAB (human T-cell lymphoma cells) and lysed freshly isolated buffy-coat mononuclear cells obtained from bovine peripheral blood, respectively. Note that these mononuclear cells are negative for endothelial cell markers CD31, Tie2 and VEGFR2.

Importantly, many clones with typical endothelial morphology by phase contrast microscopy (Figures 3A&B) had markedly different expression of endothelial receptor tyrosine kinases (RTKs) such as Tie2 and VEGFR2 (Figure 3D). In particular, clones were identified that, despite having similar monolayer morphology had either strong or weak expression of Tie2 and VEGFR2 receptors relative to BAECs as well as relative to one another (Figure 3D). All clones with endothelial morphology expressed CD31 in various amounts, showing that they were of endothelial lineage. When subsequently tested, clones positive for endothelial markers showed no expression of CD45, a pan hematopoietic marker commonly expressed on lymphocytes, mature myeloid cells and hematopoietic progenitor cells, and samples of freshly isolated mononuclear cells were negative for endothelial markers (Figure 3E).

**Figure 4:**
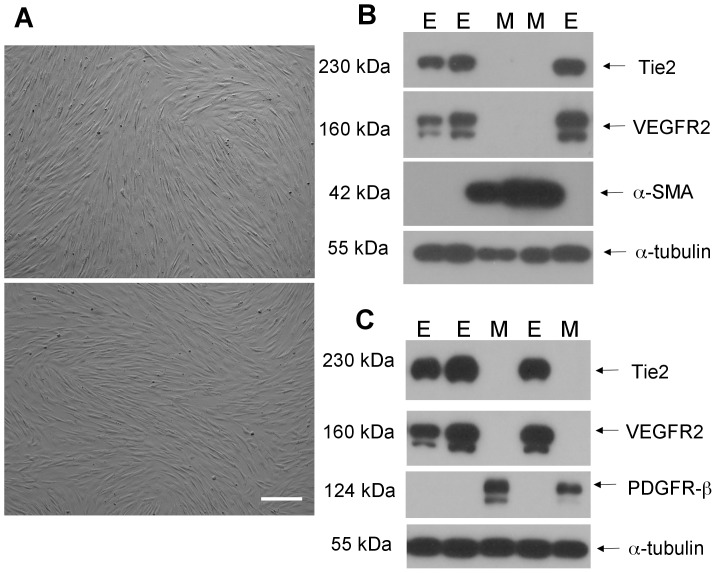
Phenotype of ‘mural cell’ clones. (**A**) Phase contrast images of two colonies arising from culture of bovine peripheral blood monocytes that do not display typical cobblestone morphology of confluent endothelial monolayers. *Scale bar  = 100 µm* (**B**) Western blot of mural clones (M) and some endothelial clones from Figure 2 (E) showing mural cells are negative for the endothelial markers Tie2 and VEGFR2, yet positive for α-smooth muscle actin (α-SMA). Conversely, the endothelial clones are positive for Tie2 and VEGFR2 and negative for α-SMA. (**C**) Western blot of mural (M) and endothelial (E) clones showing similar results with the smooth muscle/pericyte marker PDGFR-β.

Occasionally, peripheral blood mononuclear isolations gave rise to rare clones with spindle cell morphology lacking typical endothelial cobblestone appearance (Figure 4A). Western blotting revealed that such clones were negative for VEGFR2 and Tie2, yet positive for vascular smooth muscle/pericyte markers α-smooth muscle actin and PDGFR-β (Figure 4B, C), consistent with a mural cell phenotype. All endothelial clones evaluated were negative for these mural cell markers (Figure 4B, C).

### Evidence for In Vitro Angiogenesis

**Figure 5:**
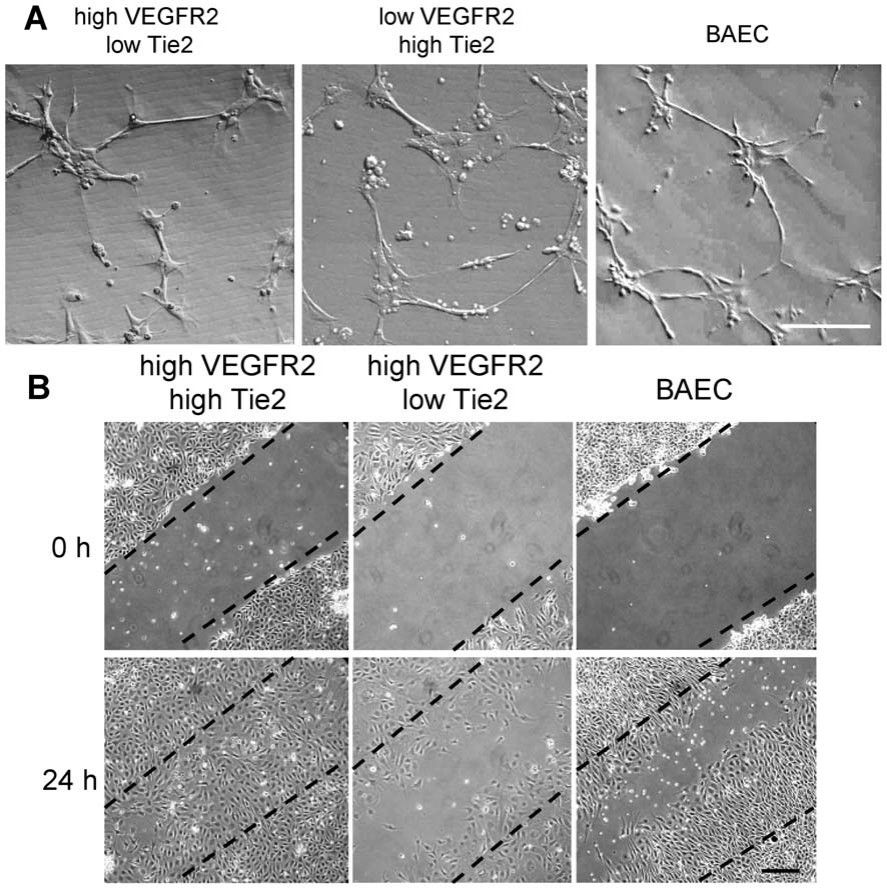
*In vitro* angiogenic capacity of clones with differential expression of Tie2/VEGFR2 receptors. (**A**) Representative images showing that clones with higher expression of either Tie2 or VEGFR2 are able to form cord-like structures on Matrigel with equal efficiency. *Scale bar  = 100 µm* (**B**) Scratch wound assay reveals that clones with higher Tie2 expression migrate and close the wound faster than cells with low Tie2 expression, although both clones have high levels of VEGFR2 expression. BAECs were used as control. *Scale bar  = 100 µm.*

Representative endothelial clones with differential RTK expression were plated on Matrigel to assess their ability to form cord-like structures. Six hours post plating, clones expressing high levels of Tie2 and low levels of VEGFR2 and vice versa formed cord-like structures in Matrigel equally well (Figure 5A). However, clones with high levels of Tie2 and low levels of VEGFR2 migrated faster to close a scratch wound in the monolayer compared to the clones that had high levels of VEGFR2 and low levels of Tie2 as well as BAECs that were used as a control (Figure 5B).

### Clones with Differential Expression of Tie2/VEGFR2 Home to Sites of Angiogenesis *in vivo*

**Figure 6:**
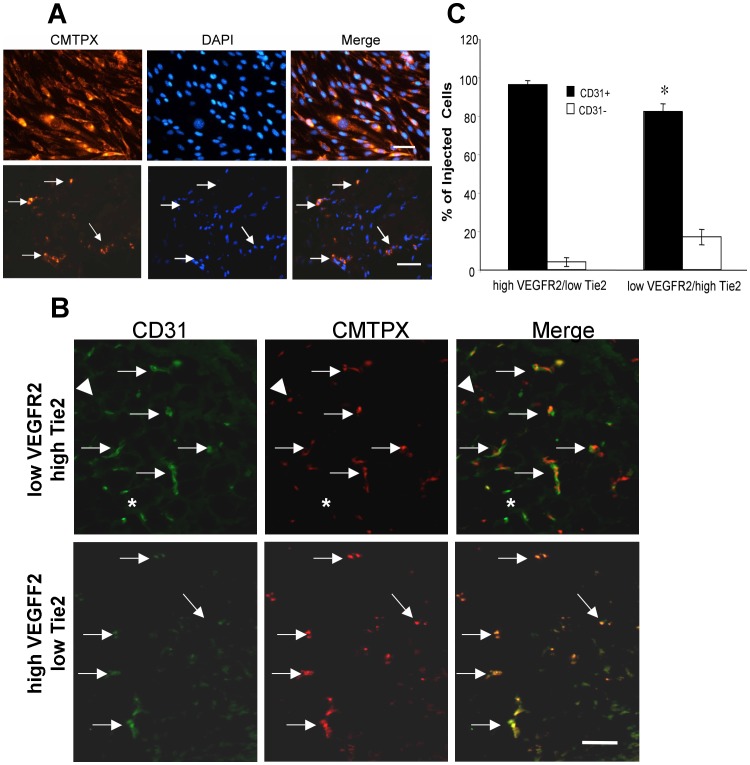
*In vivo* angiogenic capacity of clones with differential expression of Tie2/VEGFR2 receptors. (**A**) Optimization of cell tracking technique for fluorescently labeling cells using CellTracker Red CMTPX. Endothelial cells are well labeled with the tracker dye even 7 days post labeling, through multiple cell divisions *in vitro* (top panels). Bottom panels show detection of fluorescently labeled cells 7 days post tail vein injection into immune deficient mice with vascularized subcutaneous Matrigel plugs. DAPI was used as a nuclear stain in both cases. *Scale bar top panels  = 25 µm; bottom panels  = 100 µm.* (**B**) Images show cryosections from representative Matrigel plugs (low VEGFR2/high Tie2 expression in top panel, high VEGFR2/low Tie2 in bottom panel) 7 days post tail injection with CMTPX labeled endothelial clones. Infiltrating labeled cells (red) are endothelial cells as shown by co-localization of CD31 immunostaining (green), indicated by arrows. Asterisks indicate CD31 positive endothelial cells in pre-existing Matrigel plug microcirculation; arrowheads indicate CMTPX labeled CD31^−^ cells. *Scale bar  = 100 µm.* (**C**) Quantification of CD31 status of CMTPX injected cells indicates that the majority (80–95%) of injected cells are CD31+. There are significantly higher numbers of CD31^−^ injected cells in Matrigel implants from mice injected with low VEGFR2/high Tie2 expressing endothelial clones (p<0.05; N  = 3).

Clones were transiently labeled *in vitro* using CellTracker Red CMPTX *in vitro*. Optimization studies revealed that cells could be successfully labeled and maintain this dye *in vitro* for at least 7 days through several cell divisions (Figure 6A). We investigated if different mononuclear cell derived endothelial clones are able to home to sites of active angiogenesis and integrate into newly formed blood vessels in Matrigel plugs subcutaneously injected into nude mice (Figure 6A). In fact, fluorescently labeled cells (5 EPC derived clones and control BAECs) were all able to home to the Matrigel plug, regardless of their Tie2/VEGFR2 expression characteristics (Figure 6A). Moreover, these cells incorporated into growing vasculature within the Matrigel plug as evident by co-staining of CMTPX (red) cells with the endothelial marker CD31 (Figure 6B). Quantification revealed that significantly greater proportions of injected low Tie2/high VEGFR2 cells were incorporated into CD31 positive capillaries in these Matrigel plugs, compared to injected high Tie2/low VEGFR2 cells (Figure 6C). The proportion of endothelial cells negative for CMTPX label (and presumably host murine endothelium) ranged from 7 to 30% of CD31 positive cells, and was not affected by the RTK expression patterns of injected cells (no shown).

## Discussion

Here we report that endothelial clones matured from bovine blood mononuclear cells display significant differences in expression patterns of key endothelial specific RTKs. Such differential expression of Tie2 and VEGFR2 receptors in EPC derived cells may be reflected in changes in cell behavior and altered vascular phenotype during adult vasculogenesis, which could have implications for the use of EPC derived cells in tissue engineering and similar regenerative medicine, as well as impact the effectiveness of endothelial-targeting therapies. Numerous research groups have isolated and successfully grown EPCs as cell colonies ex vivo from adult peripheral and umbilical cord blood mononuclear cells [8], [18], [23], [26]–[27]. To our knowledge, there have been no other reports on isolation and identification of such cells from bovine peripheral blood. Here, we describe methodology for successful isolation of mononuclear cells from the blood of healthy adult cows that give rise to endothelial cell colonies *in vitro*. In contrast to the approaches of many research groups, we did not use FACS based identification of progenitors before the initial seeding of peripheral blood mononuclear cells [21], [28]–[30], but rather seeded the entire isolate without any pre-selection, a protocol that has been used previously with other species [9], [17]–[18]. Since large amounts of bovine blood can be obtained from the same animal, our approach allowed us to observe the wide range of phenotypes possible for endothelial cells derived from peripheral blood cell outgrowth *in vitro*, and to study in more detail the cells that can arise when subjected to specific culture conditions. We feel that this setting better represents the *in vivo* situation, where circulating adult EPCs home to the site of angiogenesis, and where the presence of natural matrix would support their adhesion and provide an ideal microenvironment for their differentiation.

In addition, many groups have focused on CFU-ECs as the *in vitro* equivalent to bone marrow derived endothelial cells [18]–[19], [31]. However, it is now apparent that endothelial colony forming cells (ECFC) give rise to cells that express endothelial markers while CFU-ECs give rise to cells expressing endothelial and hematopoietic markers [20]. This has led to the conclusion that, while some mononuclear cells in whole blood may express possible markers of undifferentiated EPCs (such as VEGRF1 and Tie2), not all cells which express such surface markers may have fully committed to an endothelial lineage [20], [32]–[34]. More importantly, ECFC but not CFU-ECs formed functional human-murine chimeric vessels when injected into mice, leading to the conclusion that ECFCs actually function as EPCs while CFU-ECs do not [20]. Based on these criteria, the late outgrowth endothelial clones we obtained and characterized in this study can be considered the bovine equivalent of these ECFCs.

The methodology used in our experiments allowed us to observe the outgrowth of both CFU-EC and ECFC from bovine blood mononuclear cells. Consistent with other reports, we found that CFU-EC did not replate well, and therefore were not the focus of further phenotype analysis. In addition, our *in vivo* studies confirmed that we in fact isolated ECFCs. This allowed us to quantify the receptor tyrosine kinases expression patterns of ECFC generated cells, and examine the impact of differences in RTKs on angiogenic behavior *in vitro.*

Our studies show that extracellular matrix does not play a significant role in efficiency of differentiation of peripheral blood mononuclear cells into endothelial cells, nor does it affect the resultant phenotype of the clones obtained. Endothelial clones derived from progenitor cells had interesting and surprising surface marker and RTK expression profiles, especially with regard to Tie2 and VEGFR2. The heterogeneity we see is consistent with the possibility that EPCs may be produced and shed by the existing vascular tree, rather than be derived directly from the bone marrow [33]. With such an origin, phenotypic differences in their resultant progeny could be due to the fact that different vascular beds are known to display heterogeneous endothelial phenotypes [35], [36]. The generation of ‘mural cell’ like colonies positive for markers of vascular smooth muscle/pericytes may reflect the differentiation potential of these EPCs, or may instead be indicative of additional populations of uncommitted vascular progenitor cells. N-cadherin is frequently expressed in vascular smooth muscle cells, and enhances their migration and survival [37]. Thus N-cadherin expression in some but far from all EPC derived clones could also reflect their differentiation along an endothelial-mural cell continuum, an issue that requires further study.

The significance of heterogeneous RTK expression in our clones was investigated through *in vitro* and *in vivo* studies. Endothelial cells with high Tie2/low VEGFR2 or the reciprocal phenotype did not display any detectable impairment in *in vitro* cord formation on Matrigel. *In vivo*, endothelial cells sprout and form a migrating column. Cells situated at the tip of the sprout (“tip cells”) sense and navigate the environment using VEGF as a major guide [38]–[41]. Cells in the stalk proliferate and follow the “tip” cell, forming a lumen [39]. This lumen formation is carefully orchestrated by VEGF distribution and it’s binding to VEGFR2 on the stalk cells [40]. Interestingly, it has been reported that Tie2 is expressed in stalk cells, but not in tip cells [40].

Endothelial clones with high Tie2 expression showed enhanced regeneration of a denuded monolayer compared to clones with low Tie2 expression, regardless of their VEGFR2 expression. There were also detectable differences in ability to home into vascularized Matrigel plugs *in vivo* between endothelial clones with differential expression of Tie2 and VEGFR2. This suggests that active sites of angiogenesis may attract variety of endothelial progenitor cells with the potential for differential expression of their receptors, such as Tie2 and VEGFR2. Their contribution could lead to newly formed blood vessels heterogeneous for these signaling molecules.

Our findings potentially have important implications in the use of anti-angiogenic therapies. Such agents targeting specific endothelial receptors, such as Tie2 or VEGFR2, are already in clinical use [42]–[45]. We have previously reported that the vasculature of many human cancers is heterogeneous for expression of VEGFR2 and Tie2 [46]–[49]. Recruitment of EPCs with the potential for differentiation into endothelium with differential expression of RTKs to angiogenic sites in solid tumors may alter the responsiveness of vessels to targeted anti-angiogenic therapy. As well, our findings have implications for vascular tissue engineering and regenerative medicine, as the functional implications of these endothelial differentiation patterns *in vivo* remains to be fully determined.
